# Identification and Functional Characterization of *Toxoneuron nigriceps* Ovarian Proteins Involved in the Early Suppression of Host Immune Response

**DOI:** 10.3390/insects13020144

**Published:** 2022-01-29

**Authors:** Rosanna Salvia, Flora Cozzolino, Carmen Scieuzo, Annalisa Grimaldi, Antonio Franco, S. Bradleigh Vinson, Maria Monti, Patrizia Falabella

**Affiliations:** 1 Department of Sciences, University of Basilicata, Via dell’Ateneo Lucano 10, 85100 Potenza, Italy; carmen.scieuzo@unibas.it (C.S.); antonio.franco@unibas.it (A.F.); 2 Spinoff XFlies s.r.l., University of Basilicata, Via dell’Ateneo Lucano 10, 85100 Potenza, Italy; 3 Department of Chemical Sciences, University of Naples Federico II, 80126 Naples, Italy; flora.cozzolino@unina.it (F.C.); montimar@unina.it (M.M.); 4 CEINGE Advanced Biotechnologies, University of Naples Federico II, 80145 Naples, Italy; 5 Department of Biotechnology and Life Science, University of Insubria, Via J. H. Dunant 3, 21100 Varese, Italy; Annalisa.Grimaldi@uninsubria.it; 6 Department of Entomology, Texas A&M University, 370 Olsen Blvd, College Station, TX 77843, USA; bvinson@tamu.edu

**Keywords:** ovarian proteins, host-parasitoid interaction, *Heliothis virescens*, *Toxoneuron nigriceps*, proteomic and transcriptomic approach

## Abstract

**Simple Summary:**

The endoparasitoid of the tobacco budworm *Heliothis virescens*, *Toxoneuron nigriceps*, has several strategies to survive, including venom and calyx fluid. This latter contains a Polydnavirus and Ovarian Proteins (OPs). They are injected into the host body together with the egg. Although much research has focused on venom protein components, little is known about OPs. OPs can disrupt the cellular immune response of the host, acting on host hemocytes, the immune cells. In this study we investigated the action of HPLC fractions derived from OPs. Two fractions caused multiple and significant changes in hemocytes, including cellular oxidative stress and actin cytoskeleton disruption, which might explain the high incidence of hemocyte death and loss of function. Furthermore, using a transcriptome and proteomic approach, we identify the proteins of the two fractions that may be involved in the observed host hemocyte alterations. Our results will help to better understand the OP components and their involvement in parasitization strategies.

**Abstract:**

The endophagous parasitoid *Toxoneuron nigriceps* (Viereck) (Hymenoptera, Braconidae) of the larval stages of the tobacco budworm *Heliothis virescens* (Fabricius) (Lepidoptera, Noctuidae) injects the egg, the venom, the calyx fluid, which includes a Polydnavirus (*T. nigriceps* BracoVirus: *Tn*BV) and the Ovarian Proteins (OPs) into the host body during oviposition. The host metabolism and immune system are disrupted prematurely shortly after parasitization by the combined action of the *Tn*BV, venom, and OPs. OPs are involved in the early suppression of host immune response, before *Tn*BV infects and expresses its genes in the host tissues. In this work, we evaluated the effect of HPLC fractions deriving from *in toto* OPs. Two fractions caused a reduction in hemocyte viability and were subsequently tested to detect changes in hemocyte morphology and functionality. The two fractions provoked severe oxidative stress and actin cytoskeleton disruption, which might explain the high rate of hemocyte mortality, loss of hemocyte functioning, and hence the host’s reduced hemocyte encapsulation ability. Moreover, through a transcriptome and proteomic approach we identify the proteins of the two fractions: eight proteins were identified that might be involved in the observed host hemocyte changes. Our findings will contribute to a better understanding of the secreted ovarian components and their role in parasitoid wasp strategy for evading host immune responses.

## 1. Introduction

Host–parasitoid interactions are among the most fascinating interactions between living organisms. Parasitoid insects have evolved very fine strategies to ensure the success of parasitization, also thanks to an arsenal of parasitic factors such as maternal factors, including the venom and the calyx fluid [1,2]. This last contains the Ovarian Proteins (OPs) and in some cases a Polydnavirus (PDV) or virus-like particles (VLP) [3]. The success of the parasitism is also assured by embryonic factors, the teratocytes [3]. It is possible to find parasitoids in several different insect orders (Diptera, Coleoptera, Lepidoptera, Trichoptera, Neuroptera) but they are very common in the Hymenoptera [4]. Parasitic factors of maternal and embryonic origin play a key role in guaranteeing the success of parasitization, in particular in the escaping of host immune defenses [5,6]. The venom is a mixture of proteins produced by the venom glands of the female parasitoid. In ectoparasitoids, the role of venom is well known, as it generally induces permanent paralysis, arrest of host development, regulation of metabolism, and inhibition of the immune response [7,8]. Information on the role of venom in the case of endoparasitoid Hymenoptera relates to fairly recent studies in some host–parasitoid systems [9,10]. In several cases, venom, together with proteins secreted by ovarian calyx cells (the OPs), may play an essential role in the success of parasitization, especially in ensuring suppression of the immune system immediately after oviposition [11,12,13]. In some biological systems, the venom also plays the important role of modifying the normal host’s development [14] or it can limit and/or suppress its reproductive potential [15,16,17]. Therefore, the venom is a crucial element for the success of the parasitization both as active part against the immune system and cooperating with the other regulation factors of maternal origin. The proteins of the ovarian calyx are synthesized in the female reproductive system of the parasitoid and injected into the hemocelic cavity of the host upon oviposition [18]. These proteins play an important role in the success of parasitization and persist in the plasma of parasitized insects, in continuous contact with circulating hemocytes, up to 96 h after oviposition [19,20]. These proteins inhibit the encapsulation process, i.e., the ability of hemocytes to form a multilayered capsule around a foreign body (such as the parasitoid egg) which is eliminated through the action of toxic substances produced by the capsule itself, including melanin [21,22]. This early protective action would serve to complement a later activity performed by the PDVs [23,24]. PDVs constitute a unique group of viruses, which exist in obligate mutualistic association with some hymenopteran wasps belonging to the family of Braconidae and Ichneumonidae [25,26]. After the parasitization, the PDV infects tissues of the host, into which it begins to express its viral genes even though it seems that there is no replication in the cells of the host [27,28,29]. The expression of the PDV genes causes important pathological symptoms that we can observe in parasitized individuals such as suppression of the immune system [5,30,31] and alteration of the endocrine equilibrium [32,33]. These alterations of the physiology and development of the host are essential for the survival of the offspring of the parasitoid [34]. The role of the teratocytes have been studied only in a restricted number of Braconidae and these studies have demonstrated that they can produce and often secrete a set of proteins with different characteristics and functions [35]. It is hypothesized that the factors secreted from this type of cells include inhibitors of the immune response, fungicide molecules, inhibitors of the juvenile hormone, protease, inhibitors of the phenoloxidase, molecules that block the production of ecdysteroids, and other factors that contribute to the nourishment of the parasitoid [35]. Thus, teratocytes are involved in the regulation of the host to obtain nutrients from its tissues and then to provide them to the parasitoid larva for its development [36]. In the host/parasitoid system *Heliothis virescens*–*Toxoneuron nigriceps*, the system object of this work, the venom, and the PDV have been well characterized, and recently we have studied the effects of the OPs on host immune system. In particular, we have shown that the OPs induce several alterations on hemocytes, including cellular oxidative stress and modifications of actin cytoskeleton, thus inducing both a loss of hemocyte functionality and cell death [21]. Overall, OPs, in combination with PDV and venom, positively contribute to *T. nigriceps* evasion of the host immune response. Here, we tested OP HPLC fractions, and for two fractions we observed the same effects shown in the previous work [21]. We identified the proteins of these two fractions by a combination of transcriptomic and proteomic approaches, resulting in the identification of eight proteins that could be involved in the alterations of the host hemocyte observed. Our results will provide insight into a more comprehensive understanding of the secreted ovarian components and the functions of the OPs associated with strategy to evade host immune response for parasitoid wasps.

## 2. Materials and Methods

### 2.1. Insect Rearing

*Toxoneuron nigriceps* parasitoids were bred as previously reported by Vinson et al. [37]; briefly, cocoons were kept at 29 ± 1 °C and adults were fed with water and honey and maintained at 25 ± 1 °C. *Heliothis virescens* larvae were reared on an artificial feeding substrate [38] (Corn Earworm Diet, Bioserve, Frenchtown, NJ, USA). Late 2- or early 3-day-old last (fifth) instar larvae of *H. virescens* were individually parasitized by *T. nigriceps*. The temperature was kept at 29 °C ± 1 °C, 70% ± 5% RH, both for non-parasitized and parasitized *H. virescens* larvae. A setup of 16 h of light and 8 h of darkness was set as photoperiod, both for host and parasitoid development.

### 2.2. Calyx Fluid Collection, Ovarian Protein Purification and RNA Extraction

Calyx fluid of two-week-old *T. nigriceps* females, containing *T. nigriceps* BracoVirus (*Tn*BV) and Ovarian Proteins (OPs), was collected as previously described [21]. Briefly, females were anesthetized on ice for 10–15 min and the whole reproductive apparatus was removed. The isolated ovaries, explanted by two females (two equivalent females), were placed in a drop of 20 µL of 1× PBS (1.3 M NaCl, 70 Mm Na_2_HPO_4_, 30 mM NaH_2_PO_4_, pH 7.2) at 4 °C and the ovarian calix were dissected to allow the flow of the calyx fluid that was subsequently purified, as previously described [21]. Approximately 80–100 ovarian calyxes were dissected for RNA extraction. TRI Reagent (Sigma, St. Louis, MO, USA) was used to extract total RNA according to the manufacturer’s instructions (Sigma, St. Louis, MO, USA). To remove any DNA contaminated, a Dnase (Turbo Dnase, Ambion Austin, TX, USA) treatment was carried out. After removing the Dnase enzyme, the RNA was purified using the Rneasy MinElute Clean-up Kit (Qiagen, Venlo, The Netherlands) according to the manufacturer’s instructions and eluted in 20 mL of RNA Storage Solution (Ambion Austin, TX, USA). Agilent 2100 Bioanalyzer (Agilent Technologies, Palo Alto, CA, USA) was used to verify the RNA integrity, while a Nanodrop ND1000 spectrophotometer was used to measure the RNA amount.

### 2.3. Toxoneuron nigriceps Protein Database Building

A custom-made protein database was created using the previously assembled and annotated *T. nigriceps* ovarian calyx transcriptome [13]. The six reading frames of the 24,759 contigs derived from the transcriptome were translated in their respective amino acid sequences using SEQtools software (http://www.seqtools.dk/ accessed on 23 November 2021), obtaining 148,554 sequences. The “*ovarian proteins T. nigriceps database*” provides useful information for the protein identification, combining transcriptomic and proteomic data.

### 2.4. HPLC Analysis of the Ovarian Proteins and Transfer on the PVDF Membrane

OPs, extracted from 40 females of *T. nigriceps*, were split by HPLC (Waters LC Module I). The sample was centrifuged at 10,000× *g* for 1 min to remove possible tissue debris, transferred onto 0.22 μm columns ULTRAFREE-MC (Millipore) and centrifuged at 3000× *g* for 1 min. The OPs were loaded on a reverse phase C18 column (Phenomenex) and eluted with a flow of 0.2 mL/min using a gradient from 5 to 100% of buffer B (Acetonitrile 70%, TFA 0.04%) on buffer A (H_2_O, TFA 0.05%) for 82 min. Fractional proteins were detected by a spectrophotometer with a wavelength of 214 nm and collected manually. The individual fractions were dried using the Speed Vac SC110 and resuspended in PBS 1X for subsequent biological assays or in water to be analyzed by SDS-PAGE, at a concentration of 2 equivalent females/μL and for subsequent proteomic analysis.

### 2.5. SDS-PAGE and In Situ Protein Digestion

Dried HPLC fractions were dissolved in loading buffer (LB1X: 2% SDS BIORAD, 50mM TRIS-HClpH6.8, 10% Glycerol SIGMA, and bromophenol blue BIORAD), fractionated by sodium dodecyl sulfate–polyacrylamide gel electrophoresis (SDS–PAGE) and stained with GelCode™ Blue Safe Protein Stain (Thermo Fisher Scientific). After destaining, two bands were cut from lanes 22 and 26, respectively. The bands were in situ hydrolyzed by trypsin as reported in [39]. Briefly, gel bands were further destained alternating washes with acetonitrile (ACN) (Honeywell, Charlotte, NC, USA), 50 mM ammonium bicarbonate (NH4HCO3) (Sigma, St. Louis, MO, USA), and cysteine residues reduced by 10 mM of dithiothreitol (Sigma, St. Louis, MO, USA), and then alkylated in 55 mM iodoacetamide (Sigma, St. Louis, MO, USA). Following extensive washings to remove the excess reagents, gel bands were then treated with trypsin. Peptide mixtures were extracted in 0.2% HCOOH and ACN and vacuum dried by a Savant SpeedVac System (Thermo Fisher Scientific, Waltham, MA, USA).

### 2.6. LC–MS/MS and Protein Identification

Each peptide mixture was dissolved in 10 μL of 0.2% HCOOH (Sigma, St. Louis, MO, USA) and analyzed by nanoLC–MS/MS on a LTQ Orbitrap mass spectrometer coupled with a nanoHPLC system (Thermo Fisher Scientific, Waltham, MA, USA). Each sample was first concentrated and desalted onto a precolumn (C18 Easy Column L = 2 cm, ID = 100 mm, NanoSeparations, Nieuwkoop, The Netherlands), and then fractionated on a C18 reverse-phase capillary column (C18 Easy Column L = 20 cm, ID = 7.5 µm, 3 µm, (NanoSeparations, Nieuwkoop, The Netherlands) by using a 250 nl/min as flow rate. The gradient used for peptide elution ranged from 10 to 60% of buffer B in 69 min. Buffers A and B have the following composition: 2% ACN LC–MS grade and 0.2% HCOOH, and 95% ACN LC–MS grade and 0.2% HCOOH, respectively. The MS/MS method was set up in a data-dependent acquisition mode, with a full scan ranging from 300 to 1800 *m*/*z* range, followed by fragmentation in CID modality of the top five ions (MS/MS scan) selected on the basis of intensity and charge state (+2, +3, +4 charges), and applying a dynamic exclusion time of 40 s. The peak list generated was uploaded in Mascot software and research was performed using the in-house database named “*ovarian proteins T. nigriceps database*”. The parameters for protein identification were set as follows: “trypsin” as enzyme allowing up to one missed cleavages, carbamidomethyl as a fixed modification, oxidation of Met and pyro-Glu at N-term if Gln is present, as variable modifications, 0.5 Da as MS/MS tolerance, and 10 ppm as peptide tolerance. Scores threshold of matches for MS/MS data was fixed at 17 for all peptides [40].

### 2.7. Collection of Hemocytes from Larvae of H. virescens

Three-day-old last instar *H. virescens* larvae were anesthetized on ice for several minutes and subsequently placed in sterilized water in 70% ethanol (*v*/*v*) and washed. Cuticle was cut near the first pair of forelegs and the pouring out hemolymph was collected with a pipette and transferred to a centrifuge tube containing 1 mL of MEAD (98 mM NaOH, 145 mM NaCl, 17 mM EDTA, 41 mM citric acid, pH 4.5) precooled solution in ice [41]. The hemolymph was centrifuged at 400× *g* for 7 min at 4 °C. A MEAD-PBS solution (1:1) was used to wash the pellet (hemocytes) twice. The hemocytes were gently resuspended in 1 mL of Grace Insect Medium (Sigma Aldrich, St. Louis, MO, USA) containing 10% fetal bovine serum (Gibco, Gaithersburg, MD, USA) and 1% antibiotic–antimycotic (Gibco, Gaithersburg, MD, USA). In 24-well culture plates (Corning Incorporated, New York, NY, USA), an amount of 1 × 10^6^ hemocyte cells per well were inoculated. The OP fractions collected as described above or 1× PBS (control) were added to the hemocytes in the culture medium and incubated at 27 °C.

### 2.8. Cells Viability 

To evaluate which HPLC fraction could have the previously observed effects by OPs on *H. virescens* hemocytes [21], a preliminary cell viability test after the treatment of hemocytes with each fraction was performed using Trypan blue staining (Sigma Aldrich, St. Louis, MO, USA). Hemocytes collected from non-parasitized larvae were treated for 2 h with each HPLC fraction (obtained from two equivalent females), and as positive control we used OPs *in toto*. Then, 1× PBS (control) was added to the hemocytes collected from healthy larvae in the culture medium and incubated at 27 °C for 24 h. Hemocytes were counted using Neubauer’s chamber under microscope (Eclipse 80i, Nikon, Tokyo, Japan) after 0.04% Trypan blue staining (Sigma Aldrich, St. Louis, MO, USA).

### 2.9. Light Microscopy Hemocyte Observations 

The only two fractions (#22 and #26) deriving from the HPLC analysis which were shown to affect the cell viability were used for subsequent analysis. Hemocytes treated for 2 h with fraction #22 (two equivalent females), fraction #26 (two equivalent females), *in toto* OPs (two equivalent females), or with 1× PBS (control) were detached from the wells, transferred on slides, and subjected to different staining methodologies: May–Grünwald GIEMSA (Sigma Aldrich, St. Louis, MO, USA), 2,7 dichlorodihydrofluorescein acetate (H_2_DCFDA) (Thermo Fisher Scientific, Waltham, MA, USA), and tetramethylrhodamine isothiocyanate (TRITC)-conjugated phalloidin (Sigma Aldrich, St. Louis, MO, USA) dyes, as previously reported [21]. Briefly, each analyzed parameter was evaluated considering five random fields in three independent replicates. Cells with alteration (vacuolization process, cytoskeletal damages, showing signs of oxidative stress) were counted as percentage of modified cells on the total number of hemocytes. Hemocytes were fixed for 10 min with 4% paraformaldehyde, washed with 1× PBS, and stained for 15 min with May–Grünwald dye followed by 30 min in 5% Giemsa stain. For H_2_DCFDA staining, cells were incubated in the dark with H_2_DCFDA 10 µM for 30 min at room temperature. For TRICT staining, TRITC-conjugated phalloidin diluted 50 µg/mL in 1% BSA (Sigma Aldrich St. Louis, MO, USA) was used, and slides were incubated for 2 h at room temperature in the dark. After each treatment, slides were washed three times with 1× PBS, and mounted with glycerol (Sigma Aldrich, St. Louis, MO, USA). For all staining methodologies, the slides were examined microscopically with Nikon Eclipse 80i equipped with a Nikon Plan Fluor 100 ×/0.5–1.3 Oil Iris objective. Five random fields of three independent replicates were recorded with a Nikon Digital Sight DS-U1 camera, and the percentage of stained/fluorescent cells was counted on the total number of cells.

### 2.10. Encapsulation Assay 

The encapsulation experiment was carried out as described in Salvia et al. [21]. Briefly, 30 Sephadex Fine G 50 (50–150 m) chromatographic beads were injected in larvae at different times (10 min, 1 h, or 3 h), after the administration of 5 µL of OPs, HPLC fractions #22 and #26, and 1× PBS (control). The encapsulation effect was observed under the microscope (Eclipse 80i, Nikon, Tokyo, Japan). As previously described [15], the time required for the formation of a full hemocyte capsule was 6 h after the injection. The chromatographic spheres attached to larval tissues were collected and counted after the longitudinal dissection of larvae. The spheres were categorized, as previously described [21] according to the degree of encapsulation as follows: 0 = unencapsulated (no hemocytes layer); 1 = capsule thickness is one or more than one layer, but less than a half of the bead radius; 2 = the capsule thickness is equal to or more than a half of the bead radius. We considered encapsulated the beads that showed case 2 after 6 h of incubation. 

### 2.11. Statistical Analysis of Data 

One-way ANOVA (analysis of variance) and Bonferroni *post hoc* tests were used in the statistical analysis, to analyze the statistical differences across all treatments. Unpaired *t*-test analysis with Welch’s correction was performed to evaluate percentage of vital hemocyte compared to control. For the encapsulation assay, we first checked that the % of recovered beads after dissection was not statistically different across the experimental groups and then we compared the percent of encapsulated beads on the number of recovered beads. Statistical analysis was performed comparing (i) all treatments, (ii) control and treated samples at the same experimental time, and (iii) comparing OP treatment and #22/#26 fraction treatment at the same experimental time. Results are presented as the mean ± SE of three independent replicates, represented by the number of wells analyzed.

## 3. Results

### 3.1. HPLC Fractions of Ovarian Proteins and Evaluation of Their Activity

**Figure 1:**
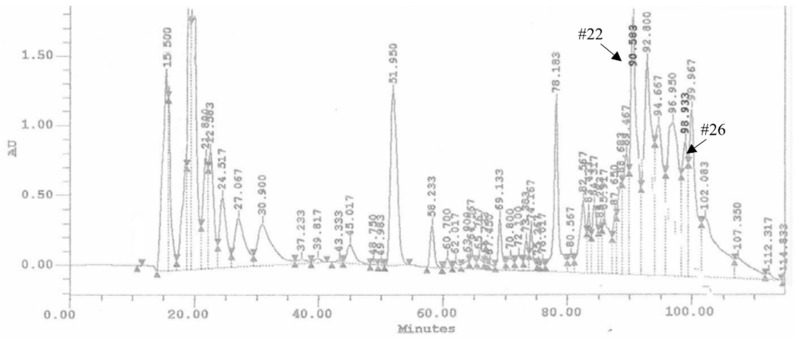
Chromatogram of HPLC analysis of OPs deriving from two equivalent females. Arrows show the retention times related to the biologically active fractions: fractions #22 and #26, respectively.

**Figure 2:**
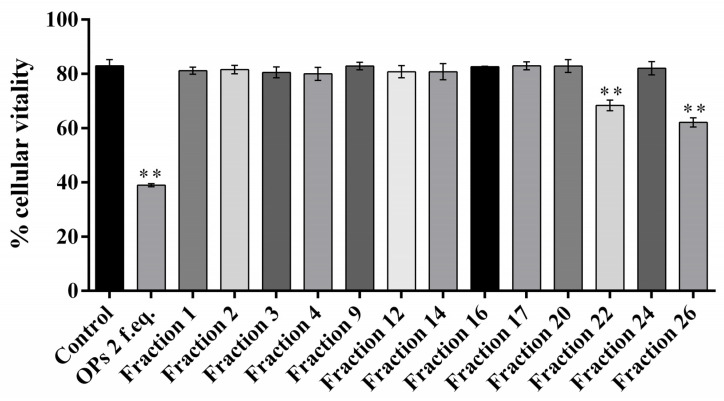
Percentage of vital hemocyte extracted from larvae incubated with 1× PBS (control), ovarian proteins (OPs), and HPLC fractions deriving from two equivalent females (2 f. eq.). Data are reported as means ± SD of *n* = 3 independent experiments. Statistical analysis was performed with unpaired *t*-test with Welch’s correction against control. The asterisk indicates significant differences (*p* value < 0.0001).

The ovarian proteins (OPs) were fractionated by HPLC using a reverse phase column C18. Figure 1 shows the chromatogram of the separation of proteins, reporting the 28 obtained fractions. For all the fractions collected, hemocytes cell viability was preliminarily evaluated. Fractions #22 and #26 showed a reduction of cell viability up to 68.34 ± 3.44% and 62.12 ± 2.89%, respectively (Figure 2). The treatment with OPs *in toto* showed 38.92 ± 0.94% of cell viability, similar to previous results [21], while other HPLC fractions did not show any effect as the control. For this reason, for subsequent analyses, we used only fractions #22 and #26, which appeared to be active and showed effects similar to those already described by OPs *in toto.* In particular, vacuolation, oxidative stress, and damage to the actin cytoskeleton were evaluated.

**Figure 3:**
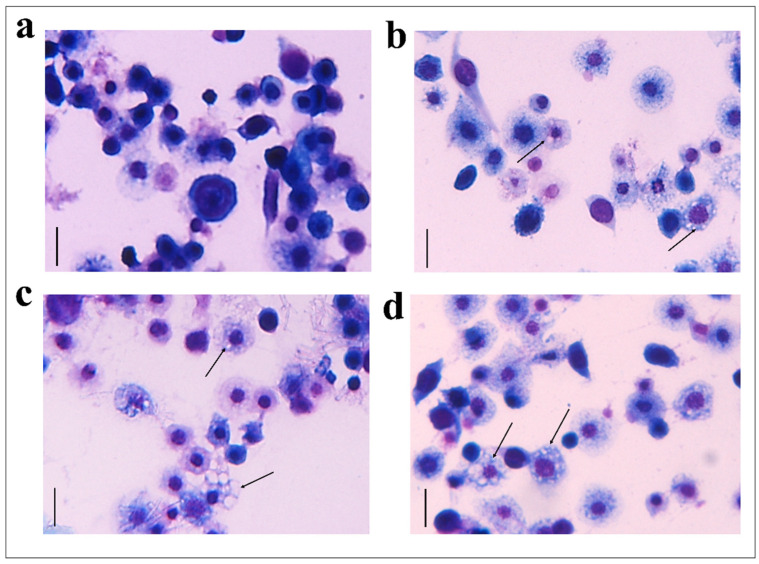
May–Grunwald–Giemsa staining of hemocytes treated with 1 X PBS (control) (**a**), with OPs deriving from two equivalent females, (**b**) fraction #22 (**c**) and fraction #26 (**d**) for 2 h stained with May–Grunwald–Giemsa dye. Scale bar 10 µm. The process of vacuolization is indicated by arrows.

**Figure 4:**
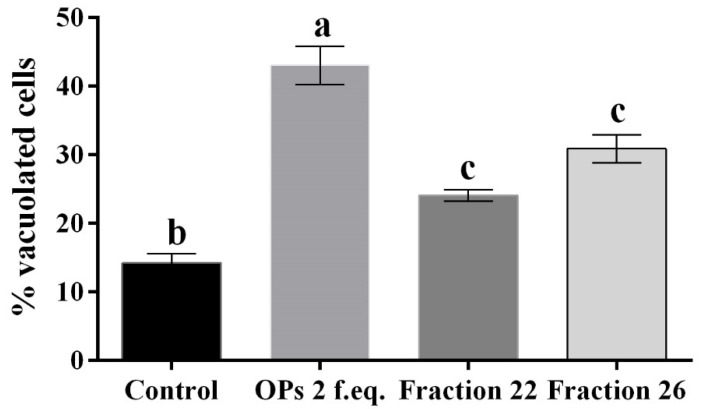
Percentage of vacuolated hemocytes, after treatment with 1X PBS (control), OPs deriving from two equivalent females (2 f. eq.) and HPLC fractions #22 and #26, observed after May–Grunwald–Giemsa staining. Data are reported as means ± SD of *n* = 3 independent experiments. Statistical analysis was performed with one-way ANOVA and Bonferroni *post hoc* test. Different letters indicate significant differences (*p* value < 0.0001).

The hemocytes were incubated for 2 h with each fraction and stained with the May–Grunwald–Giemsa dye staining that allows to detect changes of intracellular pH, often correlated with cytoplasmic vacuolization occurring after exposure to bacterial and viral pathogens or to various natural and artificial compounds [42]. Cells treated with fractions #22 and #26 showed pinkish-red acidophilic cytoplasm (Figure 3c,d) with a percentage of vacuolization major (Figure 4) to the control in which most of the cells show a not-vacuolized basophil dark blue cytoplasm (Figure 3a) (control = 14.22 ± 2.40%, OPs = 43.07 ± 4.84%, fraction #22 = 24.09 ± 1.43%, fraction #26 = 30.88 ± 3.48%).

**Figure 5:**
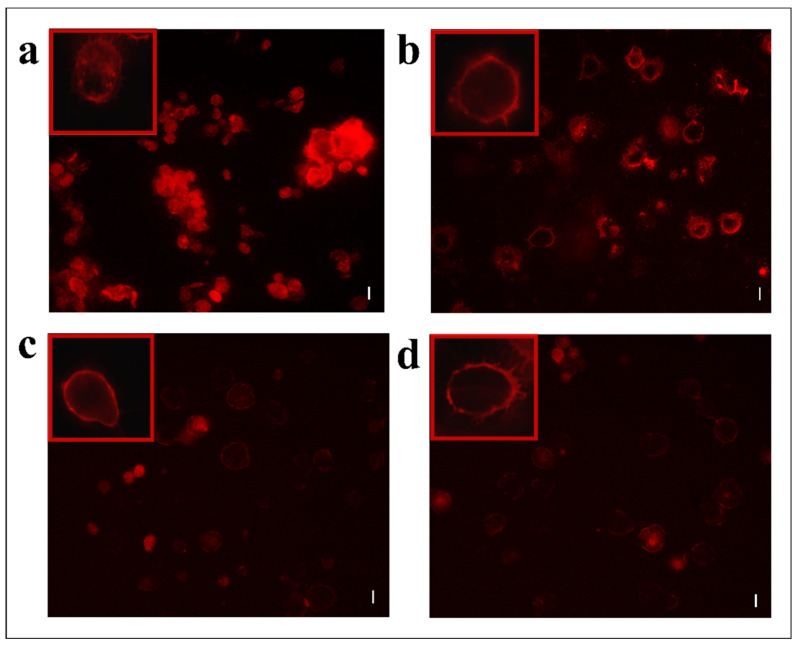
TRITC-conjugated phalloidin staining of hemocytes treated with 1 X PBS (control) (**a**) or treated with OPs deriving from two equivalent females, (**b**) fraction #22 (**c**) and fraction #26 (**d**). Scale bar 10 µm. In red boxes, enlarged cells are reported.

**Figure 6:**
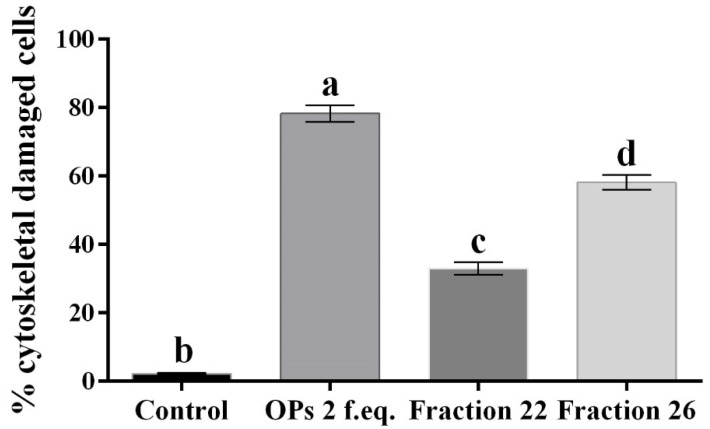
Percentage of hemocytes stained with TRITC-conjugated phalloidin showing cytoskeletal damage, after treatment with 1× PBS (control), OPs deriving from two equivalent females (2 f. eq.), HPLC fractions #22 and #26. Data are reported as mean ± SD of *n* = 3 independent experiments. Statistical analysis was performed with one-way ANOVA and Bonferroni *post hoc* test. Different letters indicate significant differences (*p* value < 0.0001).

Moreover, hemocytes, after incubation with the abovementioned fractions, were examined with the conjugated phalloidin and with the 2,7-dihydrodichlorofluorescein acetate to verify the damage of the cytoskeleton and the induction of oxidative stress, respectively. Figure 5 shows broken actin filaments close to the cell membrane in treated cells with both #22 (Figure 5c) and #26 fractions (Figure 5d), while actin filaments homogeneously distributed in control cells are observed (Figure 5a). The percentage of cells that show cytoskeletal damage on the total number of cells was equal to 78.28 ± 4.23% after OP treatment, 33.05 ± 3.21% after fraction #22 treatment, and 58.15 ± 3.64% after fraction #26 treatment (Figure 6). 

**Figure 7:**
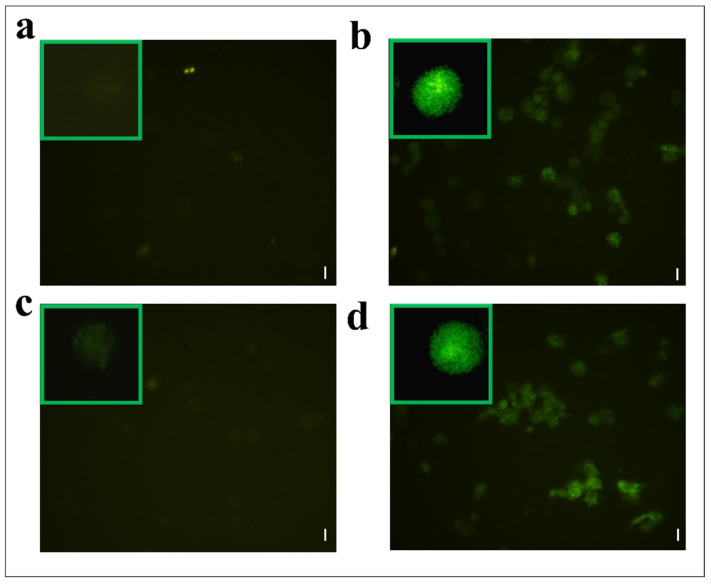
H_2_DCFDA staining of hemocytes treated with 1 X PBS (control) (**a**) or treated with OPs deriving from two equivalent females, (**b**) fraction #22 (**c**) and fraction #26 (**d**). Scale bar 10 µm. In green boxes, enlarged cells are reported.

**Figure 8:**
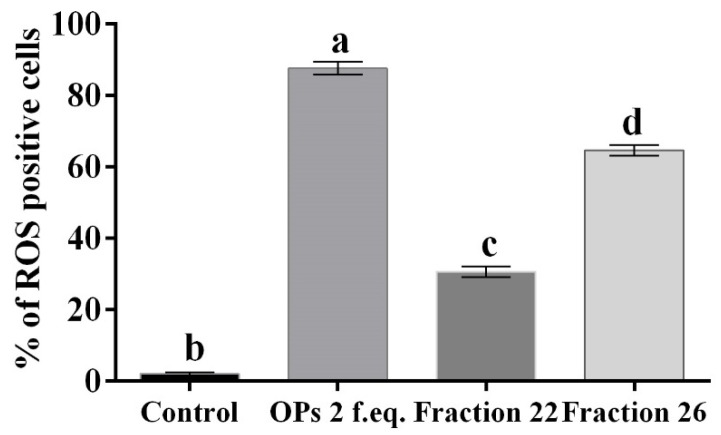
Percentage of hemocytes stained with H_2_DCFDA showing oxidative stress, after treatment with 1X PBS (control), OPs deriving from two equivalent females (2 f. eq.), HPLC fractions #22 and #26. Data are reported as mean ± SD of *n* = 3 independent experiments. Statistical analysis was performed with one-way ANOVA and Bonferroni *post hoc* test. Different letters indicate significant differences (*p* value < 0.0001).

Hemocytes were stained with 2,7 dichlorodihydrofluorescein acetate (H_2_DCFDA) after the treatment with OPs, and both fractions showed fluorescent signals indicative of oxidative stress (Figure 7c,d), while no signal was detected in control cells (Figure 7a). The percentage of hemocytes showing oxidative stress after incubation with OPs *in toto* was 87.65 ± 3.17%, 30.69 ± 2.53% after fraction #22 treatment, and 64.58 ± 2.48% after fraction #26 treatment (Figure 8). 

The encapsulation of injected chromatographic spheres employed as non-self-material was evaluated to investigate if the fractions #22 and #26 altered the capacity of the hemocytes to detect and encapsulate foreign invaders. 

**Figure 9:**
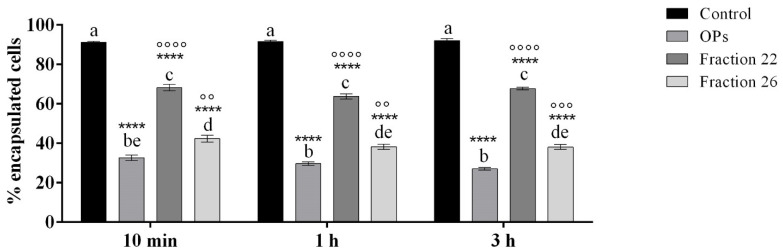
Encapsulation of chromatographic spheres extracted after 6 h from larvae treated with 1X PBS (control), OPs deriving from two equivalent females (2 f. eq.), #22 and #26 fraction at 10 min, 1 h, or 3 h before injection of spheres. Data are reported as mean ± SEM of *n* = 3 independent experiments. Statistical analysis was performed with one-way ANOVA and Bonferroni post hoc test. Different letters indicate significant differences among all treatments (*p* value < 0.0001), asterisks indicate significant differences between control and treated samples at the same experimental time (*p* value < 0.0001), and dots indicate significant differences between OP treatment,#22 and #26 fraction treatment at the same experimental time (°°°° *p* value < 0.0001, °°° *p* value < 0.001, °° *p* value < 0.01).

Figure 9 shows a strong reduction in the encapsulation capacity of hemocytes of larvae treated with HPLC fractions #22 and #26. Indeed, after the dissection of larvae, we detected a similar pattern of hemocyte encapsulation ability, regardless of the time of injection of OPs, fractions #22 and #26: percentage of encapsulation of cells treated with OPs ranged from 27.10 ± 0.71% to 32.60 ± 1.43%, percentage of encapsulation of cells treated with fraction #22 ranged from 63.81 ± 1.32% to 68.23 ± 1.61%, and percentage of encapsulation of cells treated with fraction #26 ranged from 38.15 ± 1.21% to 42.34 ± 1.68%.

### 3.2. Identification of Proteins in HPLC Fraction #22 and #26

OPs extracts were purified by RP-HPLC, and the biologically active fractions, fraction #22 and fraction #26, were subjected to a classical bottom-up proteomic procedure for protein identification.

**Figure 10:**
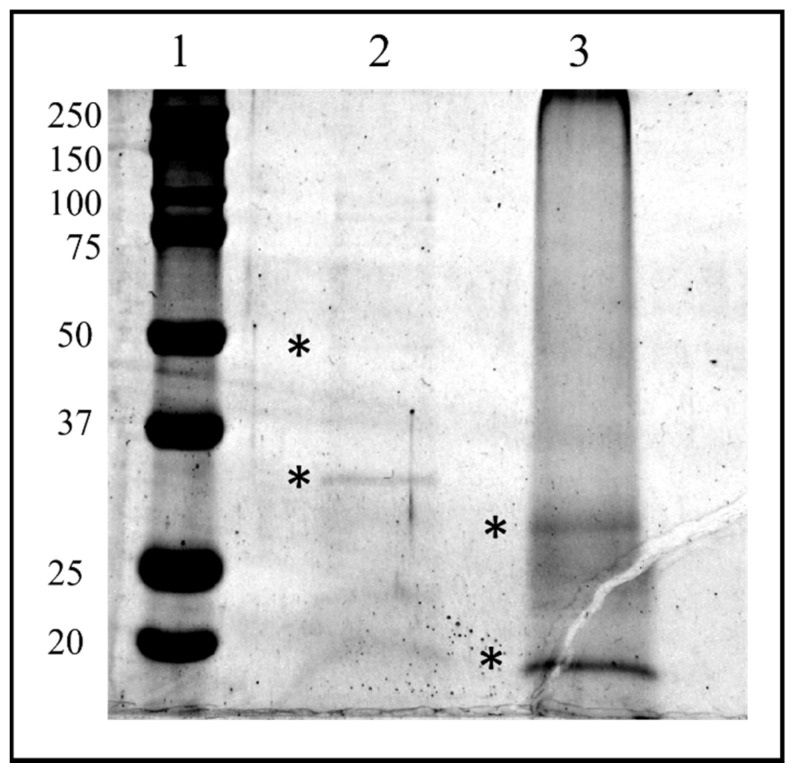
SDS-PAGE of biological active HPLC fraction Ovarian Proteins extracted from *T. nigriceps*. HPLC fraction proteins were separated on a 12.5% SDS-PAGE gel (Sigma, St. Louis, MO, USA) and stained with GelCode™ Blue Safe Protein Stain (Thermo Fisher Scientific). The bands marked with asterisks have been excised and analyzed with in situ hydrolysis digestion protocol and processed for LC/MS–MS analysis for protein identification. Lane 1: molecular weight marker (expressed in kDa) “All Blue Standards Biorad” (Biorad, Hercules, CA, USA); lane 2: proteins from HPLC fraction #22; lane 3: proteins from HPLC fraction #26.

The HPLC fractions were first dried, then resuspended in LB1X and fractionated by SDS PAGE. Two bands corresponding to the molecular weight of about 30 kDa and 50 kDa from fraction #22 and two bands corresponding to the molecular weight of about 35and 15 kDa from the lane of fraction #26 were easily visualized following colloidal Coomassie staining procedure and therefore excised from the gel (Figure 10).

The four bands were in situ hydrolyzed by trypsin, and the peptide mixtures obtained were analyzed by LC-MS/MS using the LTQ Orbitrap-XL instrument, which, in addition to the accurate measurement of the molecular weight, provided us the fragmentation spectra and then the peptide sequence of each analyzed peptide. The raw data obtained from mass spectrometry analyses were converted into mgf files and entered into the MASCOT software for protein identification procedure. The protein database employed consisted of putative protein sequences deduced from the genomic analysis of *T. nigriceps*, and present in the form of contig. Associated with each contig are reported six putative protein sequences, each for a single reading frame.

By considering the complexity of the database, due to the presence of a large number of not-expressing sequences due to the results of in silico translation, the identification procedures were carried out by applying very selective parameters: “17” as minimum acceptable threshold for peptide scores (automatically provided by MASCOT); “3” as minimum number of peptides for identifying a protein.

The amino acid sequences identified by Mascot were used to search for homologous proteins in organisms phylogenetically close to the parasitoid by means of alignment procedures using the BLASTx and the BLASTp softwares.

In Supplementary Table S1 are reported the proteins identified, including the following information: the HPLC fractions in which the proteins are found, the sequence of peptides identified, the mascot score, the *m*/*z* observed, the frame number of the transcriptomic sequence obtain with SEQtools that match with the LC-MS/MS, the start and the end of each identified peptide, the number of peptides identified for each protein, the contig code, the amino acid sequence frame (in red the peptides found by LC-MS/MS), the molecular weight of sequence, the percentage of mass peptide sequence coverage, the protein name, the query cover and the identity percentage %, and the *E*-value of candidate from BLASTp and from BLASTx alignment.

## 4. Discussion

The harmful effects of insect pests on crops represent a serious problem that affects the world food production [43]. Food demand is also expected to rise more and more due to future population growth [44]. So effective strategies for pest management, other than the indiscriminate usage of insecticides, are needed to cope with food demand. In this context, parasitoid insects could be considered powerful bio-control agents as they developed very efficient strategies to regulate the physiology of their hosts [3,7]. Specifically, maternal factors, such as Polydnaviruses, venom and Ovarian Proteins (OPs), that play a key role in the success of parasitization, could be extracted, characterized and used as molecules for biological control of pest insects [5,6,32,45]. 

Here, we study the *Toxoneuron nigriceps* OPs responsible for the functional alteration of hemocytes, such as the increase of reactive oxygen species in the cytoplasm, change of cytoplasmic pH value correlated with cytoplasmic vacuolization [46], actin cytoskeleton disruption, and increase of cellular death. Previous studies focused on the effects of *T. nigriceps* BracoVirus on ecdysteroidogenesis of *Heliothis virescens* [32,47] and the effect of venom proteins, identified through a transcriptomic and proteomic approach [13]. In endoparasitoid, the venom strongly contributes to developmental changes. Here, we report the first identification of some protein components of the *T. nigriceps* OPs integrating transcriptomic and proteomic approaches. The nanoLC-MS/MS peptide sequences of ovarian calyx HPLC fractions #22 and #26 were compared with the putative amino acid sequences of the *T. nigriceps* protein database, resulting in the identification of a total of eight different proteins. Supplementary Table S1 shows the eight identified proteins, two in the fraction #22 and six in the fraction #26. All the proteins identified showed sequence similarity with proteins of other parasitoid insects, and among them there are proteins that could have a role in the complex parasitic syndrome observed on hemocytes.

In the HPLC fraction #22, two proteins have been found, one of about 33 kDa corresponding to the contig T_C271 and annotated as an “uncharacterized protein”, and another one of about 52 kDa, corresponding to contig T_C116, annotated as a FK506-binding 59. Uncharacterized proteins are putative proteins found in a transcriptome or a genome, whose sequences correspond to an ORF, without experimental confirmation of translation [48]. Although the proteomic identification of these proteins in OPs is the experimental proof of their expression, their characterization and function are still unknown. The detection of functional conserved domains in their sequences may help to hypothesize their role. Regarding the first protein, we assume that it could be a mitotic spindle organizing 2-like, since analyzing the sequence we found the conserved domain of this protein. Proteins characterized by this domain were also identified in other Hymenoptera insects. The mitotic-spindle organizing 2-like is a protein associated with the ring of gamma-tubulin 2 and it is involved in the recruitment of mitotic centrosome proteins and complexes during the mitosis process [49]. FK506-binding, also known as heat shock protein 56 (HSP56), has several functions, including procaspase-9 and procaspase-3 activation [50].

In the HPLC fraction #26, six proteins have been found:

– Glyceraldehyde-3-phosphate dehydrogenase (GAPDH) (36 kDa, contig T_C201). It is a well-known key enzyme in glycolysis that catalyzes the first step of the pathway by converting D-glyceraldehyde 3-phosphate (G3P) into 3-phospho-D-glyceroyl phosphate [51,52]. However, it is reported in many novel cellular roles including apoptosis, tRNA export, and receptor-associated kinase [53,54,55].
– Phosphoglycerate mutase (PGAM) [56] (30 kDa, contig T_C2966) is involved in metabolism, in particular it catalyzes the reversible reaction of 3-phosphoglycerate (3-PGA) to 2-phosphoglycerate (2-PGA) in the glycolytic pathway. It is reported that in mutant mice that overexpressed Pgam2, the reactive oxygen species (ROS) was increased [57].
– Glutathione transferase (GST) (24.5 kDa, contig T_C2043) [58,59] is an enzyme that catalyzes the conjugation of glutathione (GSH) to a variety of electrophilic substances, but GST has also been shown to act as modulator of signal transduction pathways that control cell proliferation and cell death, modulating several signaling cascades [58].
– Proliferating cell nuclear antigen (PCNA) (26 kDa, contig T_C349), a cell cycle marker protein [60]. It is an essential component for eukaryotic chromosomal DNA replication and repair. The recent proteomics approaches showed that PCNA interacts with more than 100 PCNA-interacting proteins, indicating the role of PCNA in several cellular functions. Among these, it could have a possible role in apoptosis; indeed, it has been shown that apoptotic cells expressed high levels of PCNA [61].
– Apolipophorin-III (23.5 kDa, contig T_C1034, annotated as “uncharacterized protein” with a conserved domain of Apolipophorin-III superfamily) is involved in the transport of lipids [62]. However, it has been reported that in *Galleria mellonella* it plays a key role in immune response against bacteria, both Gram-negative and Gram-positive, fungi and yeasts; indeed, in *G. mellonella* larvae after immunization with Gram-negative bacteria *Escherichia coli*, Gram-positive bacteria *Micrococcus luteus*, yeast *Candida albicans*, and the filamentous fungus *Fusarium oxysporum*, the presence of this protein increased in the hemolymph, hemocytes, and fat body, enhancing the activity of antibacterial peptide such as cecropin [63,64]. The presence of this protein among the OPs could be easily explained indeed, if, on the one hand, the maternal parasitoids factors must inhibit the immune response against the parasitoid, on the other hand, they must guarantee the survival of the host, preventing the attack by other pathogens.
– Cu/Zn−superoxide dismutase (SOD1) (16.5 kDa, contig T_C1185), found in the fraction #26, could modulate the physiology of the *H. virescens*. It is a ubiquitous enzyme that catalyzes the dismutation of superoxide radicals to oxygen and hydrogen peroxide [65]. Several oxidoreductases have been found in the venom of parasitoid insects, including *T. nigriceps*, but its role in parasitization is still unknown. It could be hypothesized that SOD1 could prevent the pupation since a recent study reported that ROS production and downregulation of superoxide dismutase are required for pupation in *Bombyx mori* [66].

This study provided valuable information to deepen the role of OPs in the success of parasitization, and results could be of considerable interest for the research of new molecules to be used in biological control strategies of harmful insects in agriculture. Although this work does not provide an overall complete picture of *T. nigriceps* OPs, we searched for common proteins with venom and PDVs, finding that none of those identified in the OPs active fractions #22 and #26 overlap with the proteins previously identified in the venom [13] nor with the identified genes of *Tn*BV [27,32,67,68,69], except for a heat shock protein present in *T. nigriceps* venom. These two proteins share the putative function, but not the same dimension (70 kDa vs. 56 kDa), so we cannot consider them as corresponding proteins. The identification of specific proteins contained in the ovarian calyx, together with the previous identification of some *T. nigriceps* venom proteins (hydrolases, transferases, oxidoreductases, ligases, lyases, and isomerases) [13], strongly contributes to the deepen the mechanism underlying the host–parasitoid interactions, in which each factor contributes synergistically with the others to guarantee the success of parasitism.

## 5. Conclusions

With this research, we want to provide useful information on the possible role of specific proteins deriving from HPLC fractions of *Toxoneuron nigriceps* Ovarian Proteins (OPs). We focused the attention on the effects of these secretions on *Heliothis virescens* hemocytes: cells treated with fraction #22 and #26 partially or totally lose their vitality and their function (encapsulation process). Fraction #22 and #26 treatment increases the reactive oxygen species (ROS) in the cytoplasm and disrupts the actin cytoskeleton. With the LC-MS/MS analysis, we identified eight proteins putatively involved in the apoptosis process and ROS increasing. Our results deepen the role of OPs, that, together with other maternal factors (venom and PDV), play an active role in inhibiting the immunological response of the host, allowing the growth of the parasitoid larvae and the success of the parasitism.

## Data Availability

Data is contained within this article and the supplementary material.

## Supplementary Materials

The following supporting information can be downloaded at: https://www.mdpi.com/article/10.3390/insects13020144/s1, Table S1. Proteins identified in #22 and #26 fractions. The table reports the HPLC fractions in which the proteins are found, the sequence of peptides identified, the mascot score, the m/z observed, the frame number of the transcriptomic sequence obtained with SEQtools that match with the LC-MS/MS, the start and the end of each identified peptide, the number of peptides identified by LC-MS/MS for each protein, the contig code, the amino acid sequence frame (in red letters the peptides found by LC-MS/MS), the molecular weight of sequence, the percentage of mass peptide sequence coverage, the protein name, the query cover and the identity percentage %, and the *E*-value of candidate from BLASTp and from BLASTx alignment.
